# Effect of Incorporation of Graphene Nanoplatelets on Physicochemical, Thermal, Rheological, and Mechanical Properties of Biobased and Biodegradable Blends

**DOI:** 10.3390/polym15173622

**Published:** 2023-09-01

**Authors:** Loleny Tavares, Liliana R. Sousa, Sara Magalhães da Silva, Paulo S. Lima, J. M. Oliveira

**Affiliations:** 1 School of Design, Management and Production Technologies Northern Aveiro, University of Aveiro, Estrada do Cercal, 449, 3810-193 Oliveira de Azeméis, Portugal; lilianarcs@ua.pt (L.R.S.); sarapms@ua.pt (S.M.d.S.); plima@ua.pt (P.S.L.); 2 EMaRT Group—Emerging Materials, Research, Technology, University of Aveiro, 3810-193 Aveiro, Portugal; 3 CICECO Aveiro—Institute of Materials, University of Aveiro, Campus Universitário de Santiago, 3810-193 Aveiro, Portugal; 4 TEMA, Centre for Mechanical Technology and Automation, Mechanical Engineering Department, University of Aveiro, Campus Universitário de Santiago, 3810-193 Aveiro, Portugal

**Keywords:** nanocomposite, graphene nanoplatelets, thermoplastic starch, pine rosin, biobased blends

## Abstract

This work aimed to study the effect of the incorporation of graphene nanoplatelets (GRA 0.5% and 1% (*w*/*w*)) on the matrices of biobased polymers composed of starch-based materials (B20) and poly(butylene succinate) (PBS) using pine rosin (RES) as a compatibilizer. Three formulations were produced (B20/RES/PBS, B20/RES/PBS/GRA0.5%, and B20/RES/PBS/GRA1%), and their mechanical properties (tensile, flexural, hardness, and impact), rheological behavior, thermal properties (thermogravimetric analysis (TGA) and differential scanning calorimetry (DSC)), chemical analysis (Fourier transform infrared-attenuated total reflectance (FTIR-ATR) spectroscopy), and contact angle were evaluated. Hardness (Shore D), tensile, and flexural moduli increased, whereas elongation at break and toughness decreased as GRA content increased. FTIR studies strongly supported the existence of interactions between polymeric matrices and the large surface area of GRA. The viscosity flow curves were well fitted to the Cross-Williams-Landel-Ferry (Cross-WLF) model, and the three formulations exhibited non-Newtonian (shear-thinning) behavior. The analysis of water contact angles indicated that the formulation surfaces have hydrophilic behavior. All the samples are thermally stable, and the results of this study can be used to optimize the application of biobased graphene-based composites for applications in injection molding industries.

## 1. Introduction

Plastics are now indispensable for our way of life due to their low cost, lightweight, durability, and corrosion-resistance attributes, allied with their electrical and thermal insulation properties [1]. However, conventional plastic products, primarily composed of non-biodegradable synthetic polymers derived from petroleum, have raised significant environmental concerns given the challenges of waste management on land and in the ocean [2]. The development of biodegradable polymers (biopolymers) has been increasing in recent years as a viable solution to address these adverse environmental impacts [3]. These biodegradable materials can be made from a variety of natural feedstocks such as vegetables (such as starch, cellulose, zein, and pectin), animals, and dairy products (such as whey protein, chitosan, casein, and gelatin) [3]. Among these, thermoplastic starch (TPS) has raised great interest since starch is a renewable and inexpensive raw material, highly available in nature, and has low oxygen permeability [4,5]. However, the use of starch for the development of bioplastics presents some limitations due to its permeability to water and poor mechanical properties [6]. In order to overcome these issues, starch-based blends have been studied using aliphatic polyesters such as poly(butylene adipate-co-terephthalate) (PBAT), polycaprolactone (PCL), and polybutylene succinate (PBS) to improve their mechanical properties [7,8,9]. PBS, obtained by the condensation reaction of succinic acid with 1,4-butanediol, is the most widely commercialized biodegradable polymer due to its mechanical strength, thermal stability, toughness, and relatively low melting point, which leads to cost savings in industrial processing [10,11,12]. Since PBS is an expensive material, the use of a renewable, inexpensive polymer such as starch to substitute a partial percentage of PBS can be a viable solution to produce biobased and biodegradable products [11,13]. Starch-based polymers are hydrophilic, and PBS is a hydrophobic polymer, which makes these polymers thermodynamically immiscible, making these blends difficult to produce [14,15]. Blends based on PBS and starch have been reported in the literature [7,16,17,18]. Novamont, an Italian company specializing in bioplastics and sustainable materials, is a prominent producer of thermoplastic starch-based blends and composites; its corn starch-based products are commercialized under the brand name MATER-BI^®^ [19,20].

Pine rosin, a natural and low-cost additive, has gained interest in the bioplastics sector as a compatibilizing agent to promote miscibility between polymers [21,22]. Gum rosin and pine resin derivatives are attractive, low-cost, and renewable alternatives with great potential for developing blends with biopolymers [22]. Previous work has shown that the addition of pine resin derivatives to thermoplastic starch (TPS) led to enhanced thermal stability, causing a shift in the glass transition temperature of TPS from 45 °C to temperatures exceeding 70 °C [22]. The same authors reported that pine resin had a plasticizing effect on the polymeric matrix and acted as a compatibilizing agent, leading to an increase in toughness, impact strength, and elongation at break [23]. Studies have reported that PBS/starch blends can be improved in compatibility by reactive extrusion of isocyanate (NCO)-terminated PBS with thermoplastic starch [13], or by functionalizing the starch surface by grafting PBS (starch-g-PBS) to create strong interfacial adhesion between PBS and starch [24].

Another approach to mitigate the fragility of bioplastics is the addition of fillers, namely graphene-based materials, such as graphene nanoplatelets, graphene oxide, and reduced-graphene oxide, which are available as light nano-sized particles [25]. Graphene nanoplatelets has recently attracted major interest as a reinforcing phase in composite materials due to their low weight, excellent mechanical, electrical, and thermal properties [25,26]. In addition, graphene nanoplatelets combine large-scale production and low costs [27]. Even at low concentrations, nanoplatelets prove to be an excellent filler to improve the blend properties [28,29,30]. For example, hybrid nanocomposite blends based on biobased PBS, a biocarbon, and graphene nanoplatelets resulted in superior flexural and tensile properties of the composites [31].

To the best of our knowledge, the use of pine resin derivate as a compatibilizing agent to reinforce starch-based polymer/PBS blends and the use of graphene nanoplateletsgraphene nanoplatelets (GRA) as a filler to enhance the properties of such blends have not been reported yet. Therefore, the main objectives of this study were: (a) to produce biobased and biodegradable blends composed of a starch-based polymer (B20), PBS, and pine resin (fortified pentaerythritol (RES)); (b) to use two GRA contents (0.5% and 1% (*w*/*w*)) as a filler to reinforce the blends; and (c) to investigate the effect of GRA incorporation on the mechanical, structural, thermal, and rheological properties of the produced blends. 

## 2. Materials and Methods

### 2.1. Materials

B20, a starch-based polymer containing polybutylene adipate-co-terephthalate (PBAT), was produced using a twin-screw extruder with a melt flow index (MFI) of 4.6 g/10 min (130 °C, 10 kg) and a density of 1.28 g/cm^3^ [32]. Polybutylene succinate (PBS) with an MFI of 18 g/10 min (190 °C, 2.16 kg) and a density of 1.25 g/cm^3^ was provided by Xinjiang Blue Ridge Tunhe Polyester Co., Ltd. (Changji City, China). A pine resin derivative denominated Unik Tack P 121 resin, a fortified pentaerythritol ester of rosin with a softening point of 110 °C and an acid number of 10, was provided by United Resins (Figueira da Foz, Portugal). The graphene nanoplatelets (GrapheneBlack™ 0X) with an average particle size of 13 μm and bulk density of 0.14 g/cm^3^ were acquired from NanoXplore Inc. (Montreal, QC, Canada). The B20/PBS (50/50 wt%) blend, produced by United Biopolymers (Figueira da Foz, Portugal), has a tensile modulus of 246.34 MPa, a tensile strength of 25.63 MPa, and a Shore D hardness of 45.64, according to its product datasheet. 

### 2.2. Preparations of Blends 

In Table 1, the composition of the prepared formulations is presented. The selection of 20 wt% RES was based on a study by Pavon, et al. [33], who used pine resin derivatives at concentrations of 5, 10, and 15 wt%. The strategic decision to use a 40 wt%/40 wt% composition of B20 and PBS was primarily aimed at reducing PBS content by incorporating the economically viable starch-based polymer B20. The processing of blends B20/RES/PBS, B20/RES/PBS/GRA0.5%, and B20/RES/PBS/GRA1% was carried out using a semi-industrial co-rotating twin-screw extruder (Berstorff ZE40, Hannover, Germany). First, B20 was fed and continuously extruded at a constant screw speed of 200 rpm for 1 min at 130 °C. Then, RES was added and extruded for another 1 min, followed by the addition of PBS for 1 min. Two different GRA concentrations were considered. GRA was added to the B20/RES/PBS blend, and the mixture was continuously extruded at a constant screw speed of 200 rpm for a total of 10 min, followed by granulation. 

### 2.3. Characterization 

#### 2.3.1. Thermal Properties 

The thermal properties of formulations were evaluated through thermogravimetry analyses (TGA) and differential scanning calorimetry (DSC) using Hitachi STA300 and Shimadzu DSC-60 instruments, respectively. For TGA, samples (~10 mg) were subjected to heating, ranging from 20 to 600 °C, with a heating rate of 10 °C/min under an inert atmosphere. For DSC, the samples were subjected to two heating scans, from 25 to 110 °C and then from −40 to 200 °C. The results of the second scan (70 to 150 °C) were used to determine the melting temperature of the samples, which would be used in the injection molding process to prepare specimens for mechanical characterization. 

#### 2.3.2. Rheological Properties 

The double capillary rheometer (LCR 7002 Dynisco) was employed to assess the rheological characteristics of the formulations. The equipment was equipped with two dies of the same diameter (1 mm) but with differing lengths: one with an L/D ratio of five, and the other with an L/D ratio of 30. The evaluations were conducted at 130 °C, with an initial melting time of 5 min. The instrument was configured to operate in a consistent mode of constant speed/shear rate, encompassing a shear rate spectrum ranging from 10 to 6000 s^−1^. Both Rabinowitsch-Weissenberg [11] and Ryder-Bagley [15] corrections and the determination of melt viscosity values were accomplished using Alpha Technologies’ LAB KARS software (version 3). The melt viscosity values for each experiment were derived from an average of three trials. 

Cross–Williams–Landel–Ferry (Cross–WLF), Equation (1), was employed to describe the relationship between melt viscosity and variations in temperature and shear rate:

(1) $$\eta =\frac{\eta_{0}}{1+{\left(\frac{\eta_{0\;\cdot \;\dot{\gamma }}}{\tau^{*}}\right)}^{\left(1-n\right)}}$$

where η_0_ (Pa·s) is the reference viscosity under zero-shear-rate conditions; $\dot{\gamma }$ (s^−1^) is the wall shear rate; τ* (Pa) represents the model constant, which denotes the shear stress at the transition point between the two phases (Newtonian and non-Newtonian behavior); and *n* is the power law index.

The *η*_0_ can be determined by the WLF expressions represented in Equation (2):

(2) $$\eta_{0}=D_{1}\cdot e^{\frac{-A_{1}\left(T-\bar{T}\right)}{-A_{2}\left(T-\bar{T}\right)}}$$

where:

(3) $$A_{2}={\tilde{A}}_{2}+D_{3}\cdot P$$

(4) $$\tilde{T}=D_{2}+D_{3}\cdot P$$

where $\tilde{T}$ (K) is the material transition temperature, *D*_1_, *A*_1_, ${\tilde{A}}_{2},\;$*D*_3,_ and *D*_2_ are data-fitted coefficients. The Cross-WLF coefficients were calculated using the Solver function in MS Excel, involving the minimization of an objective function as outlined in Equation (5). This objective function encompassed the total of chi-square errors across different temperatures (i) and shear rates (j).

(5) $$the\;O=\min\sum_{i}\sum_{j}\frac{\eta {\;}_{i,\;j}^{Obs}-\eta {\;}_{i,\;j}^{Pre}}{\eta {\;}_{i,\;j}^{Pre}}$$

where, $\eta {\;}_{i,\;j}^{Obs}$ and $\eta {\;}_{i,\;j}^{Pre}$ denote the observed and predicted viscosities at a specified shear rate (${\dot{\gamma }}_{j}$) and temperature (T*_i_*), respectively. For the fitting, the A_2_ and D_3_ coefficients were fixed at values of 51.6 K and 0 K, respectively; while *n*, τ*, *D*_1_, *D*_2_, and *A*_1_ coefficients were dependent variables. 

#### 2.3.3. Fourier-Transform Infrared Spectroscopy (FTIR)

Fourier-transform infrared spectroscopy (FTIR) spectra were acquired utilizing a Bruker INVENIO S spectrometer featuring an attenuated total reflection (ATR) accessory. The measurements were conducted using a Platinum ATR (A225/Q) with a diamond crystal plate. In the analysis of each sample, the infrared spectra were acquired within the 4000–500 cm^−1^ range, employing a resolution of 4 cm^−1^ and accumulating 64 scans.

#### 2.3.4. Mechanical Testing

The pellets were molded into three types of specimens using a microinjection molding machine (Babyplast 6/10P, Cronoplast, Barcelona, Spain). The tensile and flexural tests were performed on an Autograph AG-IS universal testing machine from Shimadzu, using a 10 kN load cell and at a constant crosshead speed of 10 mm/min in accordance with ASTM D412. Tensile test specimens were produced according to EN ISO 527-2:1996 [34], type 1BA specimens. The samples were molded at 130 °C with injection and holding pressures of 110 and 100 bar, respectively. The tensile modulus was accurately determined using the noncontact video extensometer Shimadzu DVE-201, which, utilizing a universal testing machine at a consistent crosshead speed of 1 mm/min, enabled precise strain measurements without physically touching the specimen and avoiding any influence on machine deformation. For each formulation, seven specimens were taken for testing, and the average values of the tensile strength and the elasticity modulus were determined. 

Rectangular specimens were produced according to European Standard EN ISO 178: 2003 [35], measuring 80 mm in length, 10 mm in width, and a thickness of 10 mm, to conduct flexural, hardness, and impact tests. The samples were molded at 130 °C with injection and holding pressures of 120 and 110 bar, respectively. For the 2-point flexural tests, seven specimens were loaded and bent between two points separated by 64 mm, and the flexural strength and modulus were determined. The impact resistance tests were determined by the Izod test using notched specimens. A Ray Ran universal pendulum impact system was used. The specimens were subjected to impact testing after creating a notch with a depth of 2 mm and an angle of 45°. The average values of impact resistance were evaluated using at least five samples. The hardness (shore D) of the samples was measured using a digital Type D Durometer (GDS 709 J2 Teclock, Osaka, Japan) according to ASTM D 2240 [36]. 

#### 2.3.5. Contact Angle Measurement 

The contact angle measurement of the surface of rectangular injected-molded specimens (sizing 80 × 10 × 4 mm^3^) was performed using a Contact Angle Goniometer (ramé-hart instrument co., Model 190-U1, Succasunna, NJ, USA) equipped with a computer-controlled image analyzer and a video camera. Ultrapure water (PURELAB^®^ Flex 2, Elga, High Wycombe, UK) was used as a wetting liquid that was randomly deposited on the surface of specimens at 20 °C. Contact angle measurements were recorded using the Dropimage^®^ Program (Succasunna, NJ, USA). The average values of seven measurements for each drop were recorded, and the standard deviation was calculated. 

### 2.4. Morphological Analysis

The morphological structures of the samples were examined using scanning electron microscopy (SEM) with a Tescan VEGA LMS microscope (Kohoutovice, Czech Republic). The specimens were polished to remove surface defects and improve contrast, then coated with a gold-palladium alloy using an Agar Sputter Coater. The SEM analysis was conducted at an accelerating voltage of 30 keV and a magnification of 5000×.

### 2.5. Statistical Analyses

Analysis of Variance (ANOVA) was employed to assess the collected data, followed by a Tukey test for mean comparisons. The significance level was set at *p* ≤ 0.05, and these statistical analyses were performed using SAS software (version 9.3)

## 3. Results and Discussion 

### 3.1. Thermal Properties

**Figure 1:**
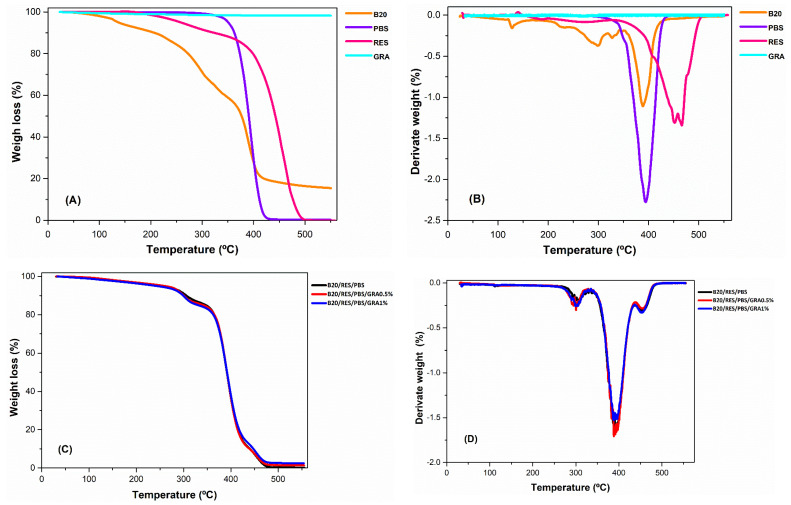
Thermogravimetric analysis (TGA) curves (**A**,**C**) and derivative thermogravimetry (DTG) thermograms (**B**,**D**) for raw materials (B20, RES, PBS, and GRA) and formulations (B20/PBS/RES, B20/PBS/RES/GRA0.5%, and B20/PBS/RES/GRA1%).

The thermal stability of the raw materials and formulations was evaluated by TGA and DTA curves (Figure 1). TGA parameters, such as degradation temperatures (T_max_) and residual mass (R_m_), are present in Table 2. For the B20, three weight loss thermal events were verified. The first event is in the range of 20 to 150 °C, with a weight loss of 6.1%, which corresponds to the removal of moisture content, specifically the release of absorbed water (Table 2). The second event from 150 to 400 °C, with a weight loss of 66.1% and a T_max_ of 388.5 °C, can be attributed to the decomposition and depolymerization of the polymeric matrices, namely the pyrolysis of starch and glycerol [37]. During the third step, at temperatures above 400 °C, the weight loss is less accentuated, explained by the decomposition of residual functional groups in the main polymers. In addition, at temperatures above 500 °C, the ashes started to form, resulting in a high-weight residue (R_m_ of 15.4%). A R_m_ of 12% was reported for biodegradable thermoplastic starch [37]. The TGA curve of PBS followed single-step degradation and decomposition of the polymer chain backbone (Figure 1B), with a T_max_ of 394.5 °C (Table 2). A T_max_ of 403.2 °C was reported by Wang, et al. [38]. For RES, two main weight-loss stages were observed. The first weight-loss stage can be attributed to the removal of adsorbed humidity and the evaporation of volatile components, including monoterpenes and sesquiterpenes, as reported by Favvas, et al. [39]. The second event can be attributed to the decomposition and degradation of the resin compounds’ structures, such as neo-abietic acid and abietic acid [39]. The TGA curve of GRA showed insignificant weight loss until 550 °C, revealing its thermal stability. In a study conducted by Wang et al. [38] the DTG curve of GRA was presented, covering a temperature range from 40 to 500 °C with minimal weight loss. However, at higher temperatures, specifically from 500 to 1000 °C, the authors observed two distinct events. The first event involved the combustion of the exfoliated GRA, which occurred at approximately 740 °C, while the second event corresponded to the combustion of the defective unexfoliated graphite fraction, which took place at temperatures > 900 °C. 

The TGA and DTG curves demonstrated that the three formulations displayed similar thermal behavior, Figure 1C,D, respectively. The primary degradation and decomposition of the polymeric matrices occurred at temperatures above 250 °C. The formulations of GRA0.5% and GRA1% exhibited similar T_max_ values, as shown in Table 2. However, the values of R_m_ differed, with an increasing trend observed as the GRA content increased. This phenomenon can be explained by the exceptional thermal stability exhibited by GRA, which persists even at elevated temperatures of up to 550 °C (Figure 1A). Based on the DTG analysis, it is evident from Figure 1D that there are three distinct bands of mass loss: the first band ranging from 250 to 340 °C, the second band spanning from 340 to 445 °C, and the third band extending from 445 to 480 °C. By comparing Figure 1D with Figure 1B, it can be concluded that the contribution of B20 is the highest in the first band, followed by both B20 and PBS in the second band, and RES exhibiting the greatest contribution in the third band.

Based on these findings, it can be inferred that the content of GRA had minimal influence on the thermal stability of the formulations. Therefore, the formulations can be processed at temperatures up to 250 °C without compromising the polymeric structure, ensuring their chemical integrity and properties.

**Figure 2:**
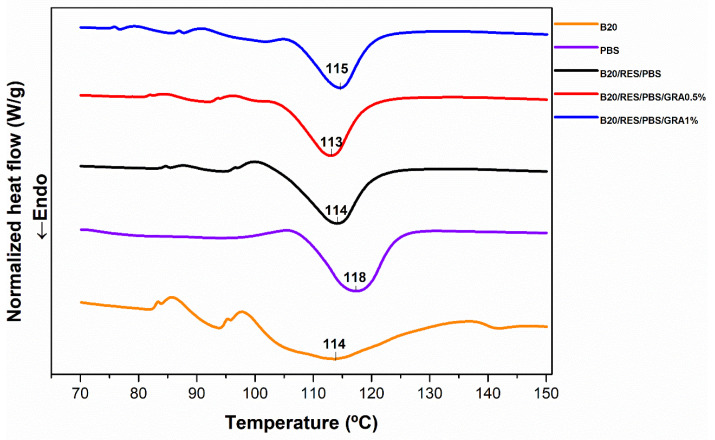
Normalized DSC curves of raw materials (B20, RES, PBS, and GRA) and formulations (B20/PBS/RES, B20/PBS/RES/GRA0.5%, and B20/PBS/RES/GRA1%).

DSC analysis was employed to determine the melting temperature (T_m_) for optimizing the injection molding conditions and producing the desired specimens. By knowing the material’s melting temperature, it is possible to optimize the injection molding conditions to ensure proper flow and filling of the mold cavity. In this sense, DSC thermograms were analyzed from 70 to 150 °C (as shown in Figure 2) to investigate the T_m_ of raw materials (B20 and PBS) as well as the formulations (B20/PBS/RES, B20/PBS/RES/GRA0.5%, and B20/PBS/RES/GRA1%). Figure 2 illustrates the endothermal peaks and corresponding T_m_ observed at approximately 113 °C for B20, 118 °C for PBS, 114 °C for B20/PBS/RES, 113 °C for B20/PBS/RES/GRA0.5%, and 115 °C for B20/PBS/RES/GRA1%. Based on these results, 130 was set as the processing temperature and applied during the injection molding process to manufacture specimens for subsequent mechanical characterization. 

### 3.2. Rheological Properties

**Figure 3:**
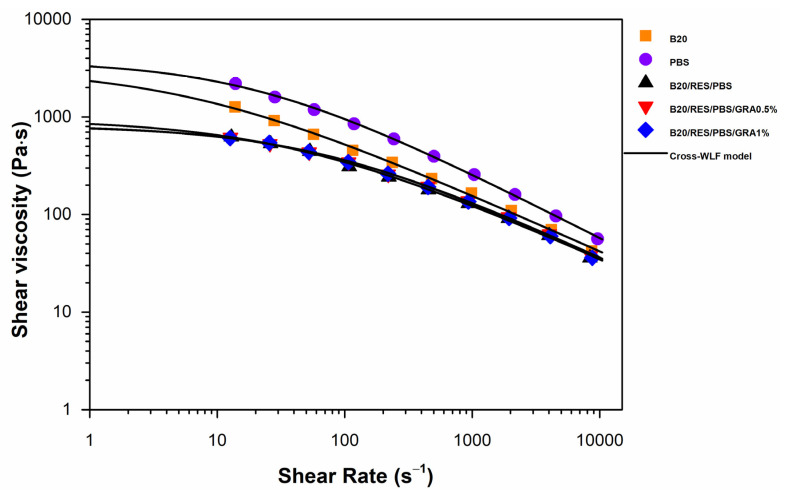
Viscosity curves for raw materials (B20 and PBS) and formulations (B20/PBS/RES, B20/PBS/RES/GRA0.5%, and B20/PBS/RES/GRA1%).

Rheology is a powerful tool for characterizing the internal structure of complex materials [40]. The influence of shear rate on viscosity was assessed by applying the Bagley and Rabinowitsch corrections to the experimental data acquired from the capillary rheometer (Figure 3), followed by fitting the experimental data using the Cross-WLF model (Table 3). The samples exhibited analogous trends, displaying a viscosity plateau at lower shear rates indicative of Newtonian behavior. Subsequently, as the shear rate increased, there was a reduction in viscosity, indicating a non-Newtonian shear-thinning (pseudoplastic) response at higher shear rates. At lower shear rates, the intermolecular entanglements between the polymer chains allow for easy slippage, leading to Newtonian behavior. In contrast, at higher shear rates, the limited time prevents the chains from disentangling fully, causing them to align along the flow direction. This alignment reduces flow resistance, resulting in shear-thinning behavior [40,41]. The results showed that with the incorporation of RES and GRA, there was a decrease in the viscosity of the blends. Lima et al. [42] evaluated the rheological behavior of polypropylene (PP)/GRA nanocomposites at 180 and detected a decrease in viscosity with the addition of GRA. The authors proposed that this reduction in viscosity could be attributed to a weak interaction between the polypropylene matrix and GRA. Li, et al. [43] conducted a rheological analysis of PP/GRA nanocomposites at 200 °C. This study examined the viscosity results for various GRA loadings ranging from 5.0 to 20.0 wt%. It was observed that at higher GRA loadings (15.0 and 20.0 wt%), the viscosity increased compared to neat PP. Conversely, at lower GRA concentrations (5.0 and 10.0 wt%), a decrease in viscosity was obtained. The researchers concluded that an essential GRA concentration for the establishment of a PP/GRA network is approximately 12.0 wt%. The rheological parameters obtained through the application of the Cross-WLF model are displayed in Table 3. The *n* values were found to be <1, which means that all formulations showed shear thinning behavior, as discussed above. PBS showed higher values of *η*_0_ and τ*, indicating higher viscosity and a greater Newtonian plateau. The results suggest that the use of RES as a compatibilizer can act as a plasticizer with a hydrolytic effect on polymer matrices, resulting in a significant decrease in viscosity. The plasticizing effect of pine resin derivatives has been reported in the literature [33]. According to Hamad, et al. [44], the low viscosity exhibited at high shear rates in the nanocomposites enables several advantages in the injection molding process. These include the ability to operate with low injection pressures, achieve high injection speeds, and reduce the overall injection cycle time. Therefore, the use of RES as a plasticizer can improve the flowability and processability of biopolymers during injection molding. Consequently, compared to B20 and PBS, the low melt viscosity of the three formulations is indicative that they can be processed under similar shear rate conditions, thereby enhancing their processability and facilitating efficient manufacturing processes.

### 3.3. Fourier-Transform Infrared Spectroscopy 

**Figure 4:**
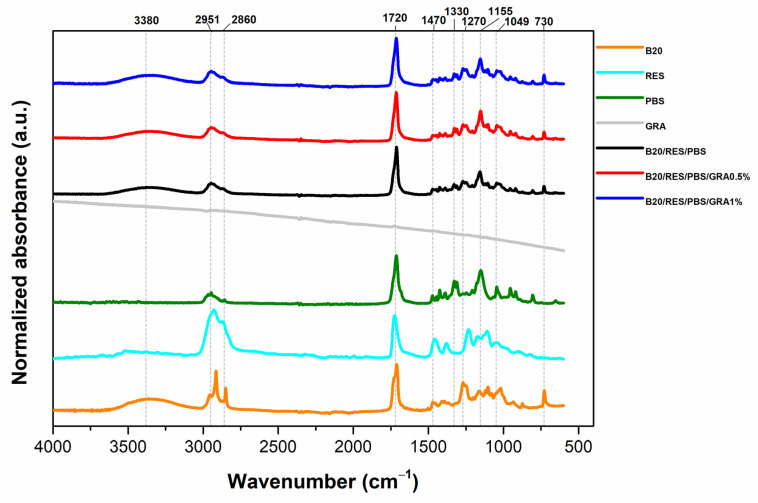
FTIR-ATR spectra of raw materials (B20, RES, PBS, and GRA) and formulations (B20/PBS/RES, B20/PBS/RES/GRA0.5%, and B20/PBS/RES/GRA1%).

FTIR-ATR analysis was performed on the raw materials and resulting formulations to examine any changes in molecular composition and physical attributes of the polymer matrices, as shown in Figure 4. The following peaks have been identified for B20, RES, PBS, B20/RES/PBS, B20/RES/PBS/GRA0.5%, and B20/RES/PBS/GRA1%: broad absorption bands between 3000 and 3600 cm^−1^ reflect the hydrolytic effect of starch on the polymeric matrix. The analysis indicated the presence of relatively weak intensity peaks around ~2951 cm^−1^ and ~2860 cm^−1^, attributed to C–H stretching. Furthermore, the existence of C=O bonds was evidenced by a peak at ~1720 cm^−1^. Additional characteristic features encompassed a peak at 1420 cm^−1^ corresponding to CH_2_ vibrations, and other peaks with comparably lower intensities were discerned at ~1330 cm^−1^ (CH bending), 1270 cm^−1^ (C–O–C stretching), 1150 cm^−1^ (C−O−C bonds), ~1049 cm^−1^ (C−O stretching), as well as 730 cm^−1^ (involving bending modes of C–H bonds within aromatic rings). For GRA, low-intensity peaks can be identified. Bilisik and Akter [45] also observed comparable tendencies, noting minor peaks at 1550 and 1650 cm^−1^, aligning with the C=O stretching vibration of the carboxylic group and the presence of skeletal graphitic carbon atoms.

Regarding formulated materials, the peaks observed for the raw materials are also observed in the formulations. However, the intensity of the broad peak at 3390 cm^−1^, attributed to the O–H stretching of starch present in B20, decreases in all formulations compared to neat B20. This can be attributed to the formation of hydrogen bonding interactions between the O–H groups of starch and the carbonyl groups of RES. Aldas, et al. [22] also reported a reduction in the intensity of the -OH group band at 3284 cm^−1^, which is associated with bound water. They attributed this reduction to the involvement of -OH groups in positive interactions between thermoplastic starch and pine resin derivatives. The intensity of the carbonyl groups (C=O at 1720 cm^−1^) increases in all formulations compared to neat RES. According to Aldas, et al. [23], the increase in intensity of this peak is due to the contribution of the ester linkages from RES. The results of FTIR spectra suggest that RES can chemically interact with polymer matrices based on B20 and PBS and that GRA can interact with these matrices through weak secondary bonds, such as van der Waals forces, dispersion forces, and hydrogen bonding.

Therefore, the formulations with GRA did not exhibit significant chemical differences and GRA did not affect the molecular structure of polymer matrices, suggesting that GRA occupied spaces in the polymeric matrix. 

### 3.4. Mechanical Properties 

The analysis of the mechanical properties was conducted to obtain a comprehensive understanding of the material’s behavior and assess its suitability for practical applications. Through the evaluation of essential metrics encompassing tensile modulus, tensile strength, elongation at break, as well as flexural modulus and strength, before the industrial application of the material, this investigation imparts crucial perspectives on its performance and inherent constraints. This knowledge improves the design and engineering processes, leading to the development of formulations that meet the specific requirements of injection molding applications, ensuring high-quality and long-lasting components. The mechanical properties are listed in Table 2. Regarding the raw materials, B20 exhibited a ductile behavior with an elongation at break of ≈1200% along with lower tensile modulus and tensile strength values. During the test, B20 exhibited remarkable necking behavior, characterized by localized thinning and elongation in specific regions of the specimen, resulting in a substantial extension before failure. PBS showed higher values of tensile modulus and strength and lower elongation at break. A similar value of tensile strength (41.5 MPa) was reported for PBS by Ou-Yang, et al. [46]. In our study, the implementation of a noncontact video extensometer allowed precise strain measurements without physically touching the specimen and avoiding any influence on machine deformation, resulting in a superior tensile modulus value of 740 MPa compared to the 554 MPa reported by these authors. For B20, due to the variability in starch sources, polymerization techniques, reinforcements, additives, and processing conditions, comparing the tensile strength of starch-based polymers can be challenging and requires careful consideration of the specific parameters involved. The behavior of the three formulations varied between the properties of B20 and PBS. A higher GRA content resulted in increased tensile modulus while also causing a reduction in elongation at break (Table 4). The incorporation of RES into the B20/PBS blend produced by United Biopolymers resulted in increased tensile strength, tensile modulus, and Shore D hardness. The increased GRA content, which has a high surface area and tensile strength, may induce strong interfacial adhesion between the GRA and B20/RES/PBS matrix. 

The decrease of elongation at break (%) is mainly due to the embedment of the graphene in the matrix, inhibiting polymer chain movement and relaxation when tension is applied, which reduces the ductility of the samples. Similar behavior was reported by Girdthep, et al. [47] who employed graphene nanoplatelets (GRA) as enhancers of properties for a blend comprising poly(butylene adipate-co-terephthalate) (PBAT) and poly(lactic acid) (PLA). For the flexural tests, B20 showed lower values of flexural modulus and strength, whereas PBS showed higher values of flexural modulus and strength. For the formulations, both moduli increased as the content of the GRA increased (Table 4). The enhancement in the composite flexural modulus is attributed to the high tensile modulus of GRA [48]. 

The results of impact resistance are presented in Table 4. For the raw materials, B20 showed a higher value of impact resistance than PBS (*p* < 0.05). Regarding the formulations, toughness decreases with the incorporation of GRA and as the content of GRA increases. The incorporation of a higher content of GRA leads to an increased surface area, and the polymer/compatibilizer adhesion is improved, resulting in a decrease in the mobility of the macromolecules (*p* < 0.05). 

From the hardness tests, B20 showed higher plastic deformation than PBS (*p* < 0.05). For the formulations, the hardness increased with the incorporation of GRA. Similar findings regarding the reinforcement of the polymer matrix’s hardness through the incorporation of GRA have been reported in other studies [49,50]. Incorporation of GRA can enhance tensile strength by constraining the mobility of polymer chains [49]. The produced blends can be used in applications that can withstand the high temperatures and pressures of the injection molding process, such as the production of automotive parts.

### 3.5. Contact Angle

**Figure 5:**
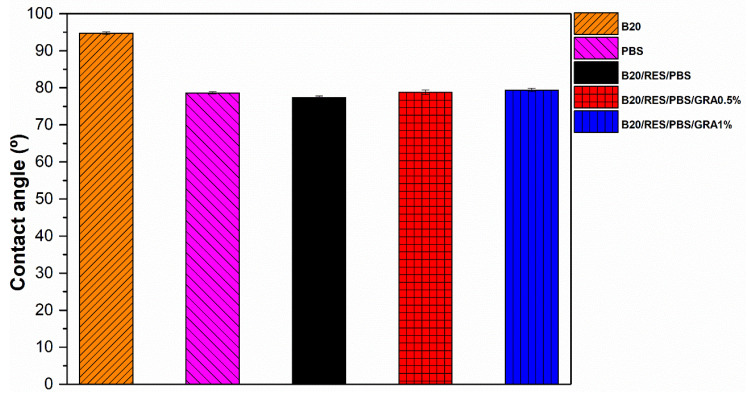
Contact angles of raw materials (B20 and PBS) and formulations (B20/PBS/RES, B20/PBS/RES/GRA0.5%, and B20/PBS/RES/GRA1%).

Water contact angles serve as indicators of the material surfaces’ hydrophobic and hydrophilic characteristics. This measurement can provide valuable insights into the surface characteristics of the materials and be realized on injected parts, allowing for an evaluation of the structural features of the final product’s surface, which offer a better understanding of the material’s performance and suitability for specific applications. The scientific community widely recognizes that a surface is classified as hydrophobic when its static water contact angle (θ) exceeds 90°, while it is deemed hydrophilic when θ is below 90°, offering a quantitative measure to evaluate water affinity and playing a critical role in diverse scientific and technological applications [51]. The values of the contact angle for the individual polymers and formulations are presented in Figure 5. B20 presented the greatest value of the contact angle, 95.07°, exhibiting a non-polar polymer surface with higher hydrophobic characteristics. This is an indicator of less attraction between its surfaces and polar water molecules. PBS and the formulations exhibited values of θ > 90°, exhibiting surfaces with hydrophilic characteristics [51]. The results for the formulations were not statistically significant (*p* > 0.5). The increase in the content of GRA in the polymeric matrices had no significant impact on the interfacial interactions and adhesion among the polymeric blends. According to Li, et al. [52], polymers with hydroxyl, phenol, and carboxylic acid groups present lower values of contact angle and surfaces with hydrophilic characteristics. The obtained results demonstrate the high suitability of the tested formulations for manufacturing hydrophilic materials specifically tailored for the injection molding industry. These materials can find valuable applications in various industries, including consumer products and industrial components, where properties such as water absorption, wettability, and moisture management are critical. 

### 3.6. Morphological Analysis

**Figure 6:**
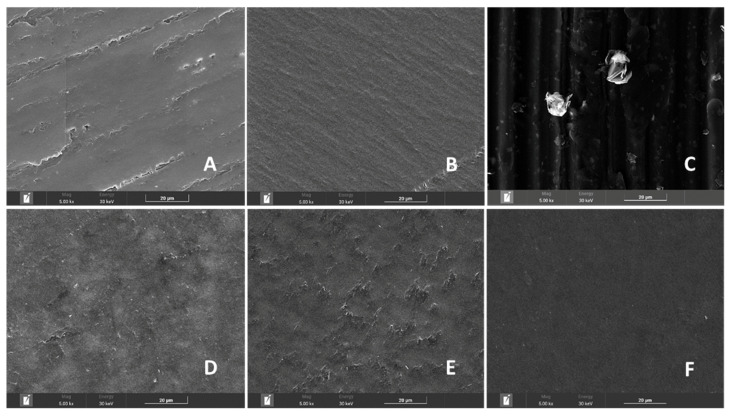
Scanning electron microscope (SEM) images of polished surfaces of specimens produced from raw materials (**A**) B20, (**B**) PBS, and (**C**) GRA and formulations (**D**) B20/RES/PBS, (**E**) B20/RES/PBS/GRA 0.5%, and (**F**) B20/RES/PBS/GRA 1%.

The effects of RES as a compatibilizer and GRA as an additive on the structure of the polymeric matrices were investigated by analyzing SEM images of polished specimen surfaces (Figure 6). The polishing step resulted in a rough surface on B20 (Figure 6A). The absence of starch granules suggests that the plasticization process was fully completed [53]. Smooth and regular surfaces were verified for the PBS sample (Figure 6B). A similar result for pure PBS was reported by Ayu et al. [7] who studied the effect of modified tapioca starch on the mechanical, thermal, and morphological properties of PBS blends for food packaging. GRA is present in the form of flakes, randomly oriented with a smooth surface (Figure 6C). 

Micrographs of the B20/RES/PB sample showed interfacial adhesion between the polymeric phases, with some small domains with empty interfaces, suggesting miscibility between the components (Figure 6D). Aldas et al. [23] reported poor interfacial adhesion between the polymeric phases in Mater-Bi-type bioplastics incorporating pentaerythritol ester of gum rosin, as evidenced by small domains with empty interfaces. The smooth surface and no obvious phase separation between the components of B20/RES/PBS/GRA1% indicate the formation of a more homogeneous phase than B20/RES/PBS/GRA0.5%, as represented in Figure 6E,F, respectively. According to the GnPs supplier, the nanoplatelets have on their edges functional groups such as ethers, carboxyls, or hydroxyls that can facilitate the GRA distribution into the matrix [42]. The results of this study suggest that the use of RES and 1% GRA is a promising approach for developing high-performance biodegradable blends with good structural integrity for use in automotive parts.

## 4. Conclusions

The purpose of this work was to evaluate the effect of two different GRA contents on the mechanical, rheological, structural, and thermal properties of polymer blends composed of starch-based materials/PBS with fortified pentaerythritol ester of rosin as a compatibilizer. The increase in GRA content has a major effect on the hardness, tensile, and flexural moduli, as well as the elongation at break and toughness. Contact angle measurements provide information about surface properties, indicating a surface with hydrophobic characteristics for GRA-based formulations. The absence of a different band formation in FTIR-ATR reveals a physical interaction rather than a chemical interaction between the formulation components. The viscosity of formulations decreased with increasing shear rate, indicating non-Newtonian shear-thinning (pseudoplastic). The mechanical performance of the produced blends, achieved at low graphene loadings, provides promising insights into the production of graphene/thermoplastic biobased composites for injection molding processes to produce automotive parts.

## Figures and Tables

**Table 1:** Compositions used in the preparation of formulations.

Formulation	Components			
	B20	RES	PBS	GRA
	%wt	%wt	%wt	%wt ^a^
B20/RES/PBS	40	20	40	-
B20/RES/PBS/GRA0.5%	40	20	40	0.5
B20/RES/PBS/GRA1%	40	20	40	1

^a^ weight percentage of the total amount based on the B20/RES/PBS formulation.

**Table 2:** Mass loss (%) during the thermogravimetric analysis as a function of the temperature ranges, maximum degradation rate (T_max_), residual mass (R_m_) for raw materials (B20, RES, PBS, and GRA) and formulations (B20/PBS/RES, B20/PBS/RES/GRA0.5%, and B20/PBS/RES/GRA1%).

Sample	Temperature Range (°C)			T_max_ (°C)	R_m_ (%)
	Up to 150	150–250	250–400		
B20	6.1	9.5	56.6	388.5	15.4
PBS	0.1	0.1	70.3	394.5	0.2
RES	0.1	4.3	16.1	466.5	0.0
GRA	0.9	0.4	0.4	46.8	98.2
B20/PBS/RES	2.4	2.9	58.1	394.4	0.2
B20/PBS/RES/GRA0.5%	1.9	2.7	58.6	388.2	1.4
B20/PBS/RES/GRA1%	2.3	3.3	56.3	388.6	2.5

**Table 3:** Cross-WLF parameters for raw materials (B20 and PBS) and formulations (B20/PBS/RES, B20/PBS/RES/GRA0.5%, and B20/PBS/RES/GRA1%).

Sample	*η*_0_ (Pa·s)	τ (Pa)	*n*
B20	2406.4	31,499.5	0.4
PBS	3403.3	87,934.1	0.3
B20/RES/PBS	800.4	54,226.5	0.4
B20/RES/PBS/GRA0.5%	778.9	62,381.9	0.4
B20/RES/PBS/GRA1%	731.6	59,969.4	0.4

**Table 4:** Mechanical properties of B20/RES/PBS, B20/RES/PBS/GRA0.5%, and B20/RES/PBS/GRA1% formulations.

Samples	Tensile Modulus (MPa)	Elongation at Break (%)	Tensile Strength (MPa)	Flexural Modulus (MPa)	Flexural Strength (MPa)	Impact Strength (kJ/m^2^)	Hardness Shore D
B20	79.74 ± 4.84 ^e^ **	1207.02 ± 50.32 ^a^	13.86 ± 1.02 ^c^	58.28 ± 4.67 ^e^	3.22 ± 0.12 ^e^	28.62 ± 1.23 ^a^	36.29 ± 0.26 ^e^
PBS	810.24 ± 10.25 ^a^	31.58 ± 3.51 ^e^	40.32 ± 3.84 ^a^	456.45 ± 5.09 ^a^	32.35 ± 2.25 ^a^	12.09 ± 0.59 ^c^	64.57 ± 0.42 ^a^
B20/RES/PBS	551.01 ± 16.27 ^d^	511.34 ± 7.45 ^b^	19.77 ± 1.84 ^b^	370.17 ± 3.54 ^d^	12.86 ± 0.11 ^d^	22.58 ± 2.05 ^b^	51.28 ± 0.56 ^d^
B20/RES/PBS/GRA0.5%	692.52 ± 11.01 ^b^	131.41 ± 4.55 ^c^	17.56 ± 1.03 ^b^	382.44 ± 4.04 ^c^	14.92 ± 0.12 ^c^	11.51 ± 0.79 ^d^	54.77 ± 0.11 ^c^
B20/RES/PBS/GRA1%	745.37 ± 15.23 ^c^	89.65 ± 5.54 ^d^	17.93 ± 1.21 ^b^	428.34 ± 6.07 ^b^	15.07 ± 0.13 ^b^	7.64 ± 0.43 ^e^	59.26 ± 0.04 ^b^

** Different letters in the same column indicate values that are significantly different (*p* < 0.05) based on Tukey’s multiple comparison test.

## Data Availability

The data presented in this study are available on request from the corresponding authors.
